# Post-Traumatic Diaphragmatic Hernia Presenting as Bowel Obstruction 12 Years After a Chest Gunshot Wound: A Rare Delayed Diagnosis

**DOI:** 10.1155/cris/2527619

**Published:** 2025-03-26

**Authors:** Papa Mamadou Faye, Ulrich Igor Mbessoh Kengne, Ousmane Thiam, Mouhamadou Laye Diop, Joël Gabin Konlack Mekontso, Mouhamed Moustapha Gueye, Seydy Ly, Amacoumba Fall, Mame Dieme Diop, Christophe Mbueda, Naomi Makam, Alpha Oumar Toure, Mamadou Cisse

**Affiliations:** ^1^Department of Surgery, Dalal Jamm National Hospital, Dakar, Senegal; ^2^Department of Surgical Oncology, Cheick Anta Diop University, Dakar, Senegal; ^3^Department of Surgery, Cheick Anta Diop University, Dakar, Senegal; ^4^Department of Medicine, South Brooklyn Health, Brooklyn, USA

**Keywords:** acute bowel obstruction, delayed post-traumatic diaphragmatic hernia, gunshot wound, surgical emergency, thoracoabdominal trauma

## Abstract

Post-traumatic diaphragmatic hernia (PTDH) is defined as the migration of intra-abdominal organs into the chest through a pathological defect in the diaphragm caused by trauma. PTDH is a rare condition, occurring in 3%–7% of all thoracoabdominal injuries. Approximately 14.6% of PTDH cases present months to years after the initial trauma. Cases of delayed PTDH complicated by bowel obstruction and perforation are exceedingly uncommon, with a reported prevalence of 0.17%–6%. In Africa, fewer than 10 cases of delayed PTDH have been documented over the past three decades. Despite the availability of published reports, there are no established practice guidelines for managing PTDH. Here, we present a case of delayed PTDH that manifested as acute bowel obstruction 12 years after a gunshot wound to the chest. The condition led to a fatal postoperative outcome. This case highlights the critical need for clinicians to consider PTDH in patients with a history of trauma presenting with acute bowel obstruction and underscores the importance of urgent surgical management to prevent fatal complications.

## 1. Introduction

Post-traumatic diaphragmatic hernia (PTDH) refers to the migration of intra-abdominal organs into the chest through a defect in the diaphragm caused by trauma [1, 2]. This occurs due to the forces of blunt or penetrating trauma, which create a sudden increase in intra-abdominal pressure exceeding 100 cmH_2_O, leading to diaphragmatic injury [3, 4]. The continuous motion of the diaphragm prevents healing, causing the defect to enlarge over time [3].

PTDH is rare, occurring in 3%–7% of thoracoabdominal injuries [5–7]. It is reported in 1%–7% of blunt abdominal trauma cases and 10%–15% of penetrating abdominal injuries [8–10]. Diaphragmatic injuries are frequently missed during the acute phase, with delayed diagnoses reported in 12%–66% of cases [1, 5, 11]. Most PTDH cases remain asymptomatic for years, only becoming apparent when complications such as bowel obstruction or perforation occur [1, 2, 12].

Fewer than 10 cases of delayed PTDH have been reported in Africa over the past three decades [13–15]. As both a rare condition and a diagnostic challenge, PTDH requires heightened clinical suspicion, particularly in patients with a history of trauma. While immediate presentations are well-documented, delayed cases complicated by bowel obstruction and perforation are particularly uncommon and clinically complex [2, 9]. Despite available literature, no practice management guidelines currently exist for this condition.

We present the case of a 43-year-old male with PTDH revealed by acute bowel obstruction, occurring 12 years after a chest gunshot wound.

## 2. Case Presentation

A 43-year-old male presented to the emergency department with a 6-day history of abdominal pain, nausea, and a 3-day history of absent bowel movements. He denied fever, weight loss, dyspnea, chronic constipation, and rectal bleeding. The patient reported a penetrating chest gunshot wound 12 years ago in another country, which was treated conservatively with a left chest tube. His medical history was otherwise unremarkable, and he was not on any medications.

On examination, the patient appeared mildly distressed, with a blood pressure of 105/62 mmHg, heart rate 102 beats per minute, temperature 36°C, respiratory rate 26 breaths per minute, and oxygen saturation of 98% on room air. Breath sounds were decreased in the lower left lung zone, and a well-healed scar was noted at the left eighth intercostal space on the midclavicular line. The abdomen was distended and tender, particularly in the left upper quadrant, but without rebound tenderness or guarding. Bowel sounds were absent, and the rectal examination was normal.

**Figure 1:**
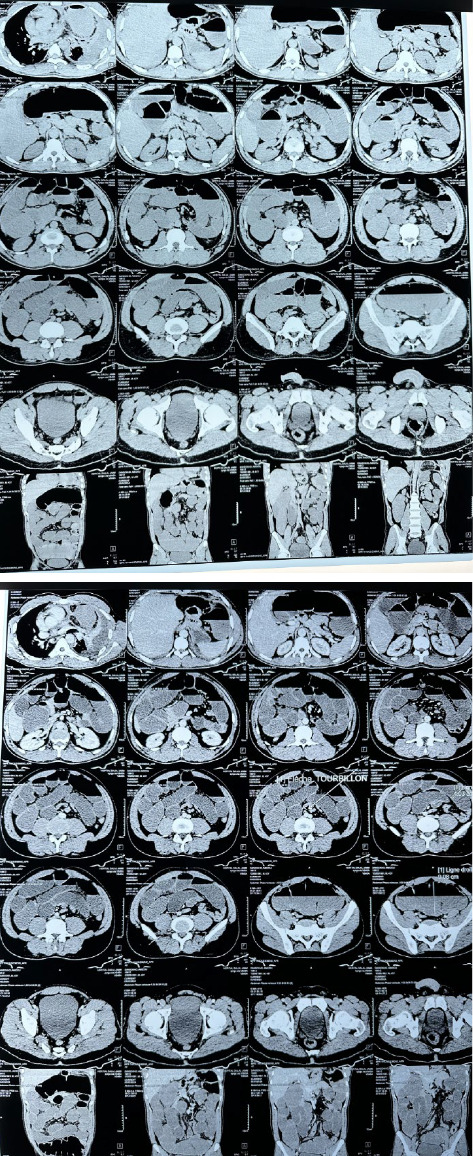
Contrast-enhanced CT scan of the abdomen and pelvis, in both axial and frontal views, showing distended bowel loops, a pattern of mesentero-axial volvulus of the colon, and a moderate peritoneal effusion.

Contrast-enhanced CT of the abdomen and pelvis revealed bowel obstruction with mesentero-axial colonic volvulus, marked bowel dilation, and a moderate peritoneal effusion (Figure 1). Blood tests indicated mild systemic inflammation (WBC 12.6 K/μL, CRP 24 mg/L), along with hyponatremia (126.1 mmol/L) and hypokalemia (K + 2.4 mmol/L). Venous blood glucose was 175 mg/dL. Urea, creatinine, and prothrombin time were normal. An electrocardiogram showed sinus tachycardia without ischemic changes.

**Figure 2:**
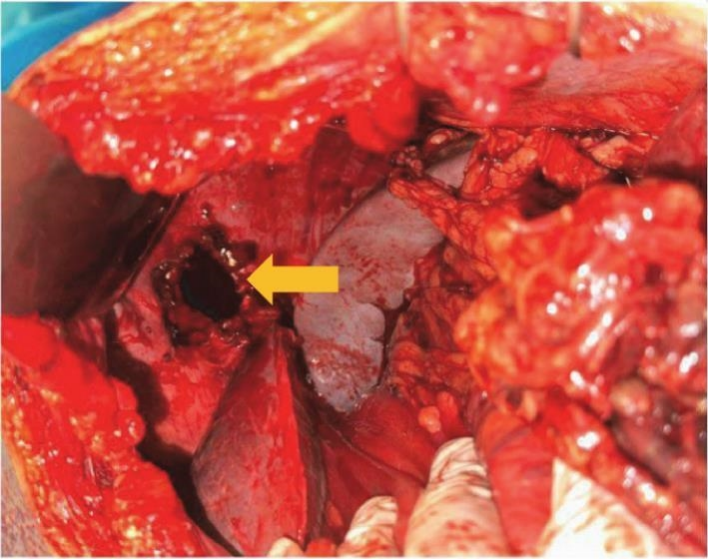
Intraoperative image of the left hemidiaphragm highlighting the diaphragmatic defect (indicated by the yellow arrow).

**Figure 3:**
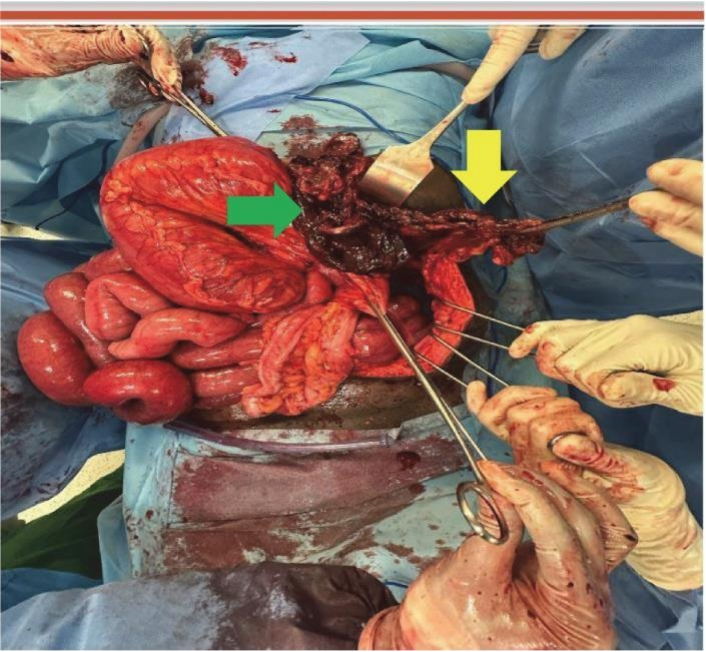
Intraoperative photograph of the bowel showing the necrotic and perforated left colic flexure (indicated by the green arrow) and the gangrenous greater omentum (indicated by the yellow arrow).

The patient underwent preoperative resuscitation with intravenous fluids, a nasogastric tube, and a Foley catheter. Thirteen hours later, he had a median exploratory laparotomy, which revealed an incarcerated bowel and omentum through a 5 × 4 cm diaphragmatic defect (Figure 2). The left colic flexure was gangrenous and perforated over 15 cm, and the greater omentum was necrotic (Figure 3). After reduction of the herniated contents, the defect was closed with interrupted 5-2 polyester sutures without reinforcement, and the gangrenous bowel segments were resected. A Bouilly–Wolkmann colostomy was performed, and the abdominal cavity was irrigated.

**Figure 4:**
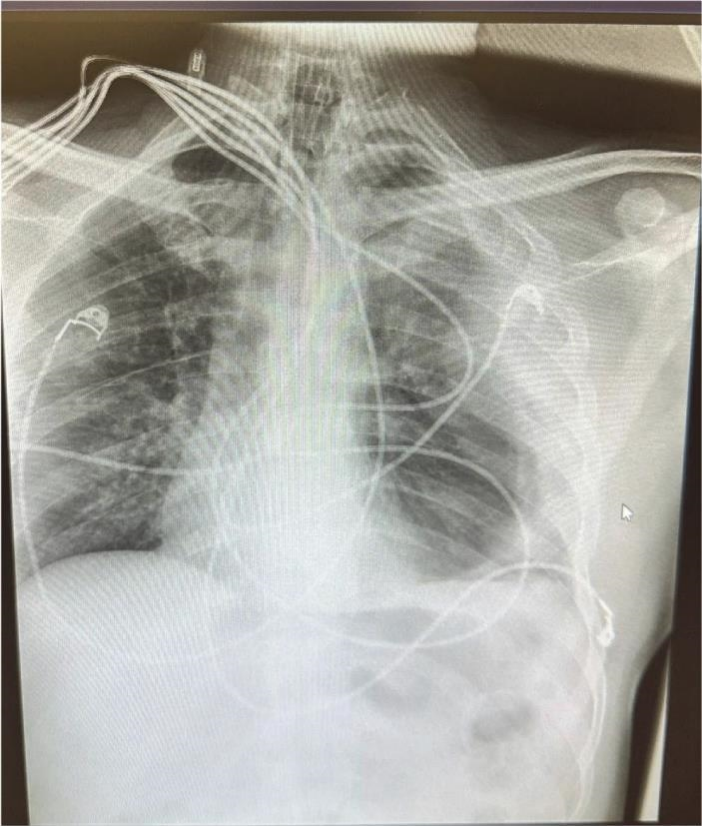
Postoperative chest radiograph on day 2 (frontal view) revealing left sided pleuropneumonia (evidenced by patchy opacities and pleural effusion).

Postoperatively, the patient was transferred to the ICU in hemodynamic instability and required sedation, intubation, and norepinephrine infusion. Despite broad-spectrum antibiotics, he developed fever from pleuropneumonia (Figure 4) on postoperative day 2, progressing to acute kidney injury and septic shock on day 4, requiring escalating pressor support. Despite aggressive management, multiorgan failure ensued and the patient expired on postoperative day 8.

## 3. Discussion

In this case, we present a rare delayed presentation of PTDH, revealed by acute bowel obstruction 12 years after a chest gunshot wound. To our knowledge, this is the first reported case in Africa of PTDH involving the herniation of the splenic colic flexure. Low PTDH documentation in Africa may reflect low incidence, underdiagnosis, or underreporting of cases. Diaphragmatic hernia was first described by Sennertus in 1541, while PTDH was first reported by Ambroise Paré in 1579 [1, 15]. The first successful repair was performed by Riofli in 1886 [1]. Blunt trauma, including falls, motor vehicle accidents, and direct blows to the chest and abdomen, is the most common cause of PTDH, accounting for 81% of cases [2, 9]. However, penetrating injuries, such as gunshot wounds, also contribute to the development of PTDH [7, 15], as seen in our patients. Yetkin, Uludag, and Citgez [5] reported that in cases of penetrating trauma to the upper abdomen and lower thorax, such as gunshot and stab wounds, physicians should be aware of the risk of latent PTDH. The high-velocity impact or penetrating nature of these injuries leads to a rapid increase in pressure gradients between the intraperitoneal and intrapleural spaces, resulting in diaphragmatic rupture [3, 11]. This defect often enlarges progressively due to the uninterrupted motion of the diaphragm.

There is a slight male predominance in reported cases with ages ranging from 16 to 71 years old [1, 7]. PTDH can present many years after the initial trauma. The frequency of delayed presentations ranges from months to decades, with the longest reported interval being 50 years [4, 9, 10]. Delayed PTDH has an estimated occurrence of 14.6% according to Yetkin, Uludag, and Citgez [5]. In our case, the condition remained undiagnosed for 12 years. The negative pressure generated during the normal respiratory cycle further plays a role in displacing intra-abdominal contents into the thoracic cavity [9, 11]. These herniations can lead to complications such as bowel obstruction, incarceration, necrosis, and perforation [10], as was observed in our patient, who presented with bowel obstruction due to incarcerated and necrotic left colic flexure and greater omentum. Bowel obstruction due to PTDH remains rare, with an incidence ranging from 0.17% to 6%, as noted by Yadav et al. [2]. More frequently, patients experience respiratory distress, but nonspecific symptoms such as shoulder and epigastric pain or finding of intrathoracic bowel sounds on auscultation can indicate diaphragmatic hernias [2, 9, 11].

Surgical reports indicate that the stomach is the most commonly herniated organ, followed by the colon, omentum, small intestine, spleen, and liver [3, 11]. PTDH is more commonly localized to the left hemidiaphragm, with studies showing that 50%–80% of cases occur on the left side, 12%–40% on the right, and 1%–9% bilaterally [1, 3, 6, 9]. This left-sided predominance is attributed to several factors: the protection provided by the liver, the tougher structure of the right diaphragmatic dome, and the weaker left hemidiaphragm at embryonic points of fusion [3, 11].

The diagnosis of PTDH is often missed on chest X-ray alone; therefore, a thoracoabdominal CT scan is considered the most appropriate diagnostic modality, with a sensitivity ranging from 71% to 100% and a specificity of 87% [1, 3]. When supplemented with MRI, sensitivity approaches nearly 100%. In our case, the clinical presentation of acute bowel obstruction and a low preoperative suspicion of PTDH led to an abdominopelvic CT scan, which failed to identify the diaphragmatic defect, underscoring the critical role of a thoracoabdominal CT scan in improving diagnostic accuracy. Furthermore, the unavailability and high cost of MRI, combined with the patient's financial constraints, rendered this imaging modality inaccessible.

Broad-spectrum antibiotics, critical for sepsis from perforated abdominal viscera, were started preoperatively. Surgical repair is essential for PTDH [1], involving reduction of incarcerated contents, hernia sac removal, pleural drainage, and closing the diaphragmatic defect [4]. This can be performed via thoracotomy, laparotomy, or laparoscopy, with or without mesh [1, 3]. While mesh repair is typically recommended for large defects (>25 cm^2^), primary suture is also safe with low morbidity, mortality, and recurrence rates [1].

Laparotomy with adhesiolysis and colonic resection was selected to minimize operative time due to the patient's hemodynamic instability. This approach is supported by Tessely et al. [1] in cases of bowel obstruction or ischemia. Surgeons may use thoracic, thoracoabdominal, or laparoscopic approaches to repair uncomplicated delayed PTDH, with the choice guided by their individual preferences and skills [9]. The absence of a thoracic surgeon in our center also influenced our decision. Pleural drainage is essential in cases of massive effusion, tension pneumothorax, or fecothorax to reduce perioperative morbidity and mortality [16], but was deferred here due to minimal pleural effusion.

The mortality rate after PTDH repair ranges from 5% to 50% depending on comorbidities [12]. The tragic course of our patient highlights the need for high clinical suspicion of PTDH after chest or abdominal trauma, effective resuscitation, and prompt surgery for acute bowel obstruction. In resource-limited settings like many African healthcare systems, delayed presentation, often due to financial constraints, contributes to poor outcomes. Our patient died on postoperative day eight from septic shock, likely due to bowel gangrene and perforation, a complication with up to 80% mortality [4].

## 4. Conclusion

This case report emphasizes the importance of considering PTDH in patients presenting with bowel obstruction, particularly those with a history of chest or abdominal trauma, regardless of the trauma's mechanism. Although rare, delayed PTDH is a potentially life-threatening condition that requires early diagnosis and urgent surgical intervention to prevent bowel gangrene. Timely surgery significantly reduces mortality risk and improves patient outcomes. Clinicians should maintain a high index of suspicion for PTDH, especially in trauma patients, to ensure effective management and reduce the likelihood of severe complications.

## Data Availability

The data that support the findings of this study are available upon request from the corresponding author. The data are not publicly available due to privacy or ethical restrictions.
